# Adaptation using IM Adapt of an evidence-based lifestyle intervention to support the management of metabolic-dysfunction associated steatotic liver disease among Hispanic/Latino adults

**DOI:** 10.3389/fpubh.2025.1628845

**Published:** 2025-07-25

**Authors:** Natalia I. Heredia, Sylvia A. Ayieko, Serena A. Rodriguez, Jessica P. Hwang, Lorna H. McNeill, Maria E. Fernandez

**Affiliations:** ^1^Center for Health Promotion and Prevention Research, School of Public Health, University of Texas Health Science Center at Houston, Houston, TX, United States; ^2^Institute for Implementation Science, UTHealth Houston, Houston, TX, United States; ^3^Department of General Internal Medicine, The University of Texas MD Anderson Cancer Center, Houston, TX, United States; ^4^Health Disparities Research, The University of Texas MD Anderson Cancer Center, Houston, TX, United States

**Keywords:** Hispanic, liver, non-alcoholic fatty liver disease, metabolic dysfunction associated steatotic liver disease, intervention development, Latino (Hispanic), intervention mapping

## Abstract

The high prevalence of metabolic-dysfunction associated steatotic liver disease (MASLD), formerly non-alcoholic fatty liver disease (NAFLD), necessitates the availability of an evidence-based intervention to manage the condition among patients to prevent severe chronic liver disease and other cardiometabolic-related illnesses. IM Adapt (Intervention Mapping for Adaptation) is a systematic approach for planning modifications in interventions to improve fit and potential effectiveness. Following this stepped approach, we conducted interviews with the target population, conducted a literature review, solicited expert advice, and created a logic model describing expected program outcomes for the new population and setting. We searched for, selected, and adapted an evidence-based intervention suitable for the target population. Given the similarities in management strategies between type 2 diabetes mellitus and MASLD (i.e., weight loss from physical activity and dietary changes), the National Diabetes Prevention Program’s Prevent T2 curriculum was selected and adapted to Hispanic/Latino patients with MASLD. We used Evidence-Based Intervention (EBI) Mapping to develop a logic model of change for the Prevent T2 curriculum, and we then compared this logic model to the one developed for our hypothetical program. Differences in content and delivery were noted, and changes were made to the curriculum. This resulted in the proposed Healthy Liver/Hígado Sano program. We also used IM Adapt to help plan for the implementation and evaluation of the program. Future testing of this intervention will determine its utility in improving the management of MASLD among Hispanic/Latino patients.

## Background

Non-alcoholic fatty liver disease (NAFLD), now called metabolic-dysfunction associated steatotic liver disease (MASLD) (1), is one of the most common causes of liver disease in the United States (US), affecting about a third (31%) of US adults (2, 3). Patients with MASLD, a condition defined by the presence of excess fat in the liver in addition to at least one of five cardiometabolic risk factors and corresponding to metabolic syndrome, experience worsening conditions with each additional cardiometabolic risk factor (4). These cardiometabolic risk factors include increased waist circumference or body mass index, prediabetes or type 2 diabetes mellitus, hypertension, elevated triglycerides, and low high density lipoprotein (5). Left unmanaged, MASLD can lead to fibrosis, cirrhosis, liver cancer, and other adverse health outcomes (6). The prevalence of MASLD is disproportionately higher among Hispanic/Latino (hereafter Hispanic) adults (38.3%) compared to adults of any other US racial/ethnic group (Non-Hispanic White: 31%, Non-Hispanic Black: 23%, Non-Hispanic Asian: 30%) (2, 3).

The mainstay evidence-based therapy for MASLD is lifestyle behavior change (including reduced dietary calorie and sugar intake, and increased physical activity) leading to weight loss (5, 7–10). Even with forthcoming fibrosis pharmacotherapy (11), and the new weight loss medications (12), lifestyle behavior change will continue to be the main pillar for disease management, especially in populations where the costs of these medications (even with insurance) make them inaccessible. Behavioral changes, such as physical activity, nutrition, and weight loss, are thus critical components of managing MASLD (5), but patients need support in achieving behavior change (13).

Evidence-based behavioral lifestyle interventions exist, though none are specifically targeted to Hispanic patients with MASLD (14). Evidence-based interventions (EBIs) adapted for new populations or cultures that adhere to the core elements increase relevance and promote engagement, often making them more effective than generic interventions (15–17). However, if adaptation is not done in a systematic way to ensure that the essential components of the intervention are preserved (18), adaptation could render the EBI less effective (19, 20). Intervention Mapping for adaptation (IM Adapt), derived from Intervention Mapping, is a process that assists program planners and public health practitioners in adapting EBIs to new contexts or populations (21), especially helping them achieve a balance between preserving essential program elements that have been key to the EBI’s effectiveness and ensuring the proposed program is acceptable to the new population or for the new setting (22).

In this study, we describe the use of IM Adapt to modify a lifestyle behavior change EBI and develop Healthy Liver/Hígado Sano, a program that aims to help Hispanic adults with MASLD make healthy lifestyle changes to prevent the progression to cirrhosis, and/or liver cancer.

## Methods

**Figure 1:**
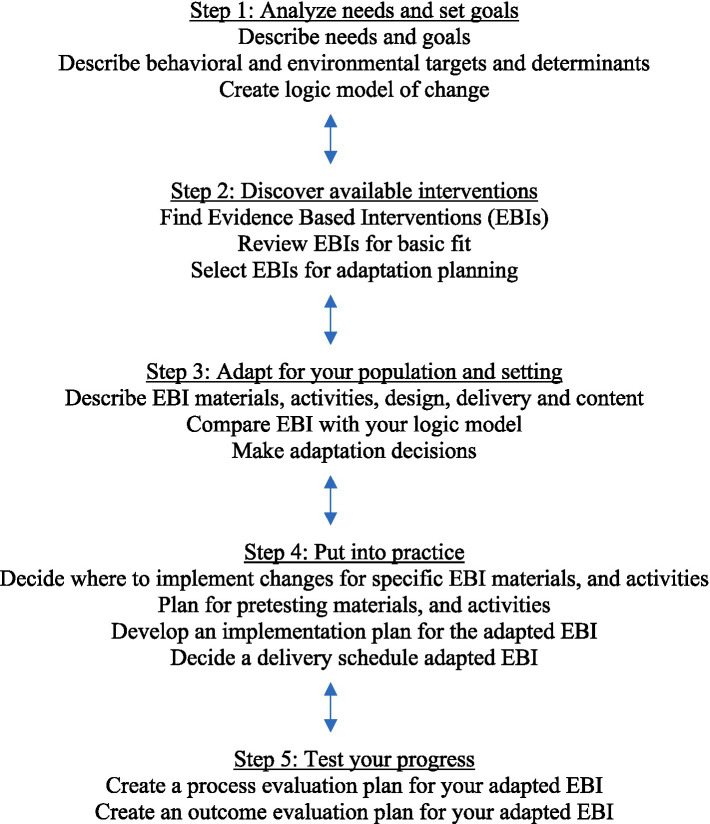
IM Adapt process (25).

IM Adapt includes five steps that help program planners understand and document the needs of a new population, identify the most effective and relevant existing EBIs, and facilitate decision-making for adaptation. The process uses a systematic approach to compare the logic model of the existing intervention with the logic model of the change needed in the current context or population (23). The steps include: (1) document needs and build a logic model of change; (2) identify available interventions; (3) adapt intervention for target population and setting; (4) plan for implementation by pretesting materials, activities, and the delivery schedule; and (5) create process and outcome evaluation plans for the adapted EBI (Figure 1). IM Adapt is an iterative process that moves back and forth between steps (24). An online IM Adapt tool developed with funding from the National Cancer Institute (25) guides decision makers through the steps and the specific tasks associated with each step. We used this tool to identify and adapt an intervention, ultimately creating the Healthy Liver/Hígado Sano program.

### Step 1. Document needs and build a logic model of change

Step 1 includes three tasks: (1a) describe patient population needs; (1b) describe desired changes; and (1c) create a logic model of change.

#### 1a. Describe needs of the patient population

To understand the needs of Hispanic adults with MASLD, we conducted a literature review and interviews with patients diagnosed with MASLD. The literature review included studies describing perceptions about MASLD, behaviors related to MASLD management, and barriers to MASLD management. Our search included studies that discussed weight perceptions, lifestyle behaviors (i.e., physical activity, healthy eating), and weight loss among adult patients with MASLD specifically, individuals with obesity in general, and individuals with other metabolic-related conditions (e.g., type 2 diabetes).

In addition, we conducted semi-structured interviews with Hispanic patients diagnosed with MASLD recruited from a Federally Qualified Health Center (FQHC) located in a predominantly Hispanic neighborhood in Houston, TX. Methods are described elsewhere in detail (13). In short, in partnership with the FQHC, we recruited participants using convenience sampling, screened them for eligibility (MASLD diagnosis and 18 years or older), and obtained informed consent. Twelve participants were enrolled. Interviews were conducted via phone or a video conferencing platform and explored participants’ experiences of being diagnosed with and managing MASLD (13), in addition to expectations of a program focused on MASLD management for Hispanic adults, and perceived usefulness of such a program. Participants were asked about behaviors, environmental conditions, and determinants. Topics also included motivations for participating in a MASLD management program, preferences for program delivery, desire for social support, and possible barriers to consistent attendance in the program. Interviews were recorded with participant permission, transcribed, and translated to English. Qualitative analysts created a codebook, conducted independent coding of the transcripts, and developed consensus of the codes and overarching themes. Analyses were conducted using Atlas.ti (26).

#### 1b. Describe desired changes in behaviors and behavioral determinants

The overall goal of the program was to ensure participants could manage MASLD to prevent cirrhosis, liver cancer, and other adverse health outcomes associated with the disease, such as cardiovascular disease. In Task 1b, we identified the specific behaviors participants would need to perform in order to achieve the goal. In addition, we identified the behavioral determinants that should be targeted by the program to ensure participants could perform those behaviors. Behavioral determinants include specific cognitive factors, often theoretical constructs, that must change in order for a person to perform a behavior (22). The needs assessment findings informed our list of behaviors and behavioral determinants.

#### 1c. Create a logic model of change

Following the literature review, interviews with patients with MASLD, statement of the behavioral outcome, and description of behavioral and environmental determinants, we created a logic model of change. The logic model connected the behavioral determinants, related target behaviors, and environmental agents, to the overall program goals.

### Step 2. Search for evidence-based interventions

In Step 2, we searched for existing EBIs and assessed the basic fit of the EBIs, including their alignment with our health problem, health-promoting behaviors, and priority population. To identify EBIs, we reviewed the Community Guide (27), the published literature, and Evidence-Based Cancer Control Programs (28) for EBIs that focused on the following: (1) prevention of adverse health outcomes through lifestyle modification; (2) MASLD management; (3) increased physical activity for weight loss or weight maintenance; and/or (4) healthy dietary habits for weight loss. Importantly, we searched for EBIs that included physical activity or dietary habits for weight loss even if they did not focus exclusively on MASLD because the overall goal of our intervention is achieving weight loss through lifestyle modifications. For example, interventions that included physical activity or dietary changes for individuals with obesity or type 2 diabetes were included in our search. In assessing the basic fit of EBIs, we examined program materials to assess their cultural appropriateness for Hispanic adults and identified opportunities for tailoring, if needed. In addition, we assessed interventions based on IM Adapt Step 1 findings. Finally, after reviewing available EBIs, we selected one program for adaptation that most closely fit our needs and program goals.

### Step 3. Assess fit and plan adaptations

In Step 3, the IM Adapt tool guided the careful examination and documentation of the materials, content, and logic of the existing program. The process, called Evidence Based Intervention (EBI) Mapping (29), helps identify the existing program design, activities, and delivery channels as well as the primary areas of intervention content (30). In EBI Mapping, program planners utilize processes from Intervention Mapping (22) but apply the systematic process retrospectively to develop a logic model of the EBI (29). Following the thorough description of the existing intervention and documenting it in a logic model, we compared the existing EBI’s logic model with the logic model we developed in *Step 1*. This enabled us to make a systematic and detailed assessment of the intervention’s fit with our new population and setting, as well as the behavioral outcomes and targeted determinants. Based on this comparison, we decided what to add, delete, modify, and maintain from the existing EBI. The IM Adapt online tool provided guidance through this process with prompts and questions. The adaptation decisions include an assessment of needed delivery changes, design features, and cultural fit. We carefully examined which components of the original program were essential and should be retained. As part of the cultural adaptation, we considered “surface structure” (e.g., language, images, and food choices) as well as “deep structure” elements including the cultural, social, and environmental influences on health behaviors (e.g., the importance of family and gender roles) (31, 32). The process of identifying the needed adaptations was iterative and involved multiple team members reviewing the existing EBI. We discussed the adaptations needed to ensure that all the planned adaptations were incorporated into the new curriculum.

At the conclusion of Step 3, we listed the essential program components to be retained and program goals to be addressed in the adaptation. We also detailed the delivery channels, design features, and cultural adaptations that needed to be applied to the original EBI.

### Step 4. Make adaptations

In Step 4, we adapted the program elements identified in Step 3 by preparing the design document for adaptation. The design document outlined the specific changes that were needed in the final document. After confirming that all the features had been incorporated into the new curriculum, we worked with the production team (i.e., graphic designer and editor) to produce proto-type materials. The adaptation process involved two bi-lingual speakers, who ensured the existing Spanish curriculum to be adapted was still relevant and appropriate for the target population. We then pre-tested the materials with a few patients at the FQHC who had participated in the MASLD interviews and asked them what they thought about the materials. These individuals reviewed the adapted materials to determine if they appeared acceptable, what they liked and disliked, and if additional changes were needed. The research team discussed the proposed revisions, refined the content, and incorporated the feedback. Once all materials were finalized, we pilot-tested the intervention with a sample from the target population (i.e., Hispanic patients diagnosed with MASLD) who were recruited from a primary care practice at a FQHC. We also adapted the implementation plan (tasks that must be completed to implement the program) from the existing EBI.

### Step 5. Plan for evaluation

In the final IM Adapt step, we developed all evaluation plans and measures for pilot testing the curriculum. We outlined a plan for data collection during the pilot testing. This included developing a process evaluation plan, identifying an outcome evaluation design, writing evaluation questions, identifying scales and measures, planning for data collection, and developing an analysis plan.

## Results

### Step 1. Document needs and build a logic model of change

#### 1a. Needs assessment

Based on the literature review, evidence from published studies suggested a need for programs or other ways to facilitate and support patients with MASLD in achieving weight loss through lifestyle modifications in dietary behaviors and physical activity (5, 33). At the time of this needs assessment (2021), there were different types of diets and physical activity being tested in patients with MASLD, with no conclusive indication that any one diet or exercise regimen was better than the other. The most important factor for MASLD management was that lifestyle changes could be maintained and would lead to weight loss (34, 35).

Preliminary data from our work indicated that while Hispanic patients with MASLD had accurate self-perceptions about their weight, they did not practice healthy eating behaviors (e.g., had inadequate fruit/vegetable consumption) or adhere to recommended physical activity guidelines (36). Barriers to physical activity included feeling lazy or lacking the time and place to exercise. Barriers to eating healthy foods included perceptions related to cost, poor taste, and lack of time and skills to cook (30). In general, these noted barriers are similar to barriers reported by adults without MASLD; therefore, existing behavioral lifestyle interventions available for the general population can be used to address barriers experienced by patients with MASLD with adaptations to address deep and surface structure and include MASLD-specific education (30). Moreover, ongoing work at the time that was later published indicated a dearth of behavioral lifestyle interventions for patients with MASLD, especially culturally-relevant ones for Hispanic patients in particular (14).

In in-depth interviews with Hispanic women with MASLD, most participants indicated that a program focused on changing diet and physical activity behavior for weight loss would be helpful and useful. Specifically, interview participants described the following program features and characteristics as important: (1) covering a variety of topics related to liver health and lifestyle; (2) being offered in an in-person, group setting; (3) feasible to attend and flexible. One participant suggested that such a program would help them learn more about their health condition, MASLD: “…It would be useful for me if they gave more information about this—regarding the liver, so that I could understand better” (Participant 3).

Related to the delivery setting and modality, one participant noted “It would help me a lot because I would feel more motivated if I was in a group. I would not feel like I’m the only one with this condition” (Participant, 4). Another participant felt that being in a group setting would motivate them to keep losing weight (Participant 5). When asked about the delivery of the proposed MASLD intervention, most participants preferred in-person sessions although they were not opposed to a virtual setting. For example, one participant stated: “I prefer it that way ……—in person because that way you can see—you can get better explanations, in person and everything” (Participant 3). Another participant stated “No, I’d like it to be in person. You take advantage of it virtually if you know about computers and can see—but I say it’s better in person” (Participant 4). One participant highlighted the benefits of in-person sessions based on what she heard about other support groups attended by acquaintances.

“*The truth is that—look, in person because I have heard about people who have like a club of diabetic people, and they go there and talk, and there is somebody there giving them advice. And then I have a friend, for example, who has breast cancer. she used to invite me to some lessons … where they talk about the same, diet, lifestyle, exercise, and everything they can do to stay healthier, to help their body. I would like to have a group like that one. I would like to have those lessons and go there personally and be able to talk because I feel that it is like more—doing it in person than doing it online— like more motivating*” (Participant 10).

However, some preferred participating in virtual, but synchronous sessions because of their convenience, “Yes, it would be easy—to me, it would be convenient that way” (Participant 2) and because of distance “Well, if it is too far, virtually. If it is not so far, in person” (Participant 1). However, participants had different preferences for attending the sessions based on their work schedules and daily activities. Those who worked during the day preferred weekend or evening programs. Among participants with school-age children, some preferred early mornings while others preferred early afternoons (12 pm-3 pm).

Study participants reported some potential barriers to consistent program attendance including lack of time, transportation challenges, weather conditions, and a lack of motivation. For instance, one participant said, “So if there is bad weather, well, no, I’m not going to leave my house” (Participant 10). One emphasized that that distance and parking could be barriers.

*“……If it is too far away and there is no parking available, or the parking is too expensive—there is nowhere to park, or it costs fifteen dollars to park in the garage. I will not pay that amount. And then, if it is far away from my house, if it is an hour away or forty-five minutes, I will not go either*” (Participant 6).

Regarding strategies to encourage participants to attend sessions, participants mentioned incentives such as gift cards and gym memberships. Others felt that having sessions where participants share their progress would be encouraging. “I think that—like exercising there, I think, or testimonies too” (Participant 9). However, the empowerment that results from increased understanding of the disease and the knowledge and skills to improve their health seemed to be most important as illustrated below.

*“I think the best incentive for whoever it is--to become aware of the disease—there are no other things that just—that help us to be healthy. But there are people that maybe they do want—maybe gifts—I do not know. It depends on each person. But I think that for me, the most important thing is just to maintain my health, my fat and sugar levels and all that. That’s more than enough for me, but there are people who maybe need another kind of motivation”* (Participant 8).

“*Sometimes an incentive does help. I mean, if they tell you that they are going to give you a gift. But I think that the most important thing—the most important thing is that what they explain to you there, what you are learning—that’s more important than getting a gift”* (Participant 4).

Overall, patients with MASLD felt that a program that would help them manage their condition would be helpful if it provided more information on MASLD and lifestyle changes including physical activity and healthy eating. Several patients acknowledged that having the program delivered in person would be beneficial, emphasizing the support from other participants as an important component.

#### 1b. Behavioral and environmental targets and determinants

The program’s overall goal was to help Hispanic participants to lose weight (primary goal), reduce liver steatotis and/or liver stiffness, and manage MASLD to prevent its long-term consequences, including cirrhosis, liver cancer, or other adverse health outcomes. Therefore, we targeted health-promoting behaviors and environmental factors related to weight loss to support this overall goal and stated the objectives related to both. The primary behavioral objectives were to eat a healthy diet (focusing on energy-balance and calories) and work up to 150 minutes of at least moderate-intensity physical activity per week, along with limiting alcohol consumption. These behavioral objectives aligned with the minimal specificity in practice guidelines at the time for lifestyle behaviors in patients with MASLD (53), as well as national guidelines for adults (54). To achieve these objectives, we identified determinants influencing these behaviors in Hispanic populations. Given the flexibility of IM Adapt, we explored multiple behavioral theories, such as the theory of planned behavior (37, 38), socio-cognitive theory (39, 40), and the health belief model (41–43). We then selected constructs that influenced the specific behaviors. For example, to eat healthy food, participants need to know what foods to consume and those to avoid (knowledge), and learn how to choose and cook healthy foods (skills). We also considered determinants relevant to Hispanic culture, such as *familismo,* and reviewed determinants influencing dietary change and regular physical activity from previous interventions related to obesity, type 2 diabetes, or weight loss. We also wanted to encourage individuals to limit alcohol use, aligning with practice guidelines at the time and current guidelines (5, 53). We selected determinants such as outcome expectations, overcoming barriers, and knowledge as they were relevant for our population.

#### 1c. A logic model of change

**Figure 2:**
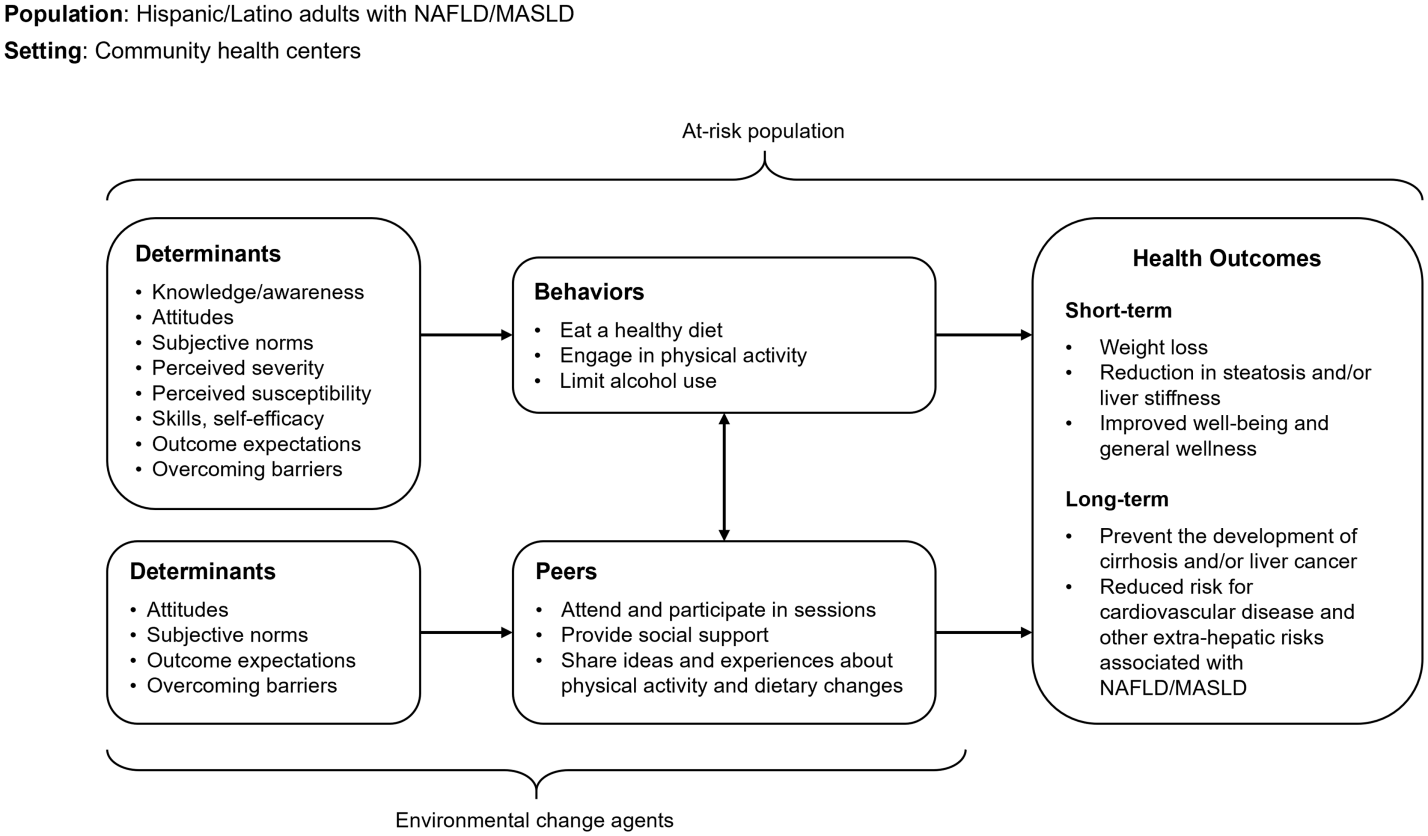
IM Adapt-logic model of change.

Figure 2 depicts the logic model of change.

### Step 2. Search for evidence-based interventions

After reviewing the interventions aimed at improving lifestyle behaviors or achieving weight loss, we selected the Diabetes Prevention Program (DPP) as the EBI that was most appropriate for our target health problem and population. The DPP is the gold standard in lifestyle interventions, focusing on physical activity and dietary behaviors to achieve weight loss (44, 45). Investigators with expertise with DPP provided feedback on the efficacy, effectiveness, and cultural relevance of the program and recommended adapting the Centers for Disease Control and Prevention (CDC) DPP curriculum, *Prevent T2* [*Type-2 Diabetes* (46)]*. Prevent T2* is an EBI with a good basic fit to the behavioral objectives of the proposed project (47, 48).

We also selected *Prevent T2* because of the curriculum features it offered, including strategies for weight loss and practical steps for physical activity and healthy diets. *Prevent T2* is offered in both English and Spanish, which served our needs, and the Spanish version incorporates examples of foods and physical activity types that are relevant for the Hispanic population. *Prevent T2* is delivered in a group setting and facilitated by a lifestyle coach. The participant curriculum is easy to comprehend with the Spanish curriculum written at the 5th-grade reading level and the English curriculum written at the 6th-grade reading level. Our team concluded that this freely available curriculum included relevant elements, messages, and images that could be adapted to develop the MASLD-specific intervention. While the DPP has been adapted for many populations and settings (47), at the time it had been adapted for Hispanic patients with MASLD.

### Step 3. Assess fit and plan adaptations

Given we were unable to locate a logic model of change for the EBI at the time of adaptation (only located evaluation logic models with inputs, outputs, etc.), we used EBI Mapping to apply the systematic process of Intervention Mapping retrospectively. Using the available intervention curriculum (Participant Guide and the Lifestyle Coaching Guide) and published studies describing the intervention, we assessed the content and activities for program components in *Prevent T2*. Then, we developed a logic model of change that identified the methods being used and the determinants, behaviors, and health outcomes being targeted. We also examined the design and delivery channels, focusing on the intervention’s look and feel, cultural elements, language, and literacy level.

The modules in the Participant Guide are available in English and Spanish, include user friendly navigation, with the Spanish version including pictures reflecting the target audience. The Lifestyle Coach Training Guide has modules that include notes to the coach, prompts and guidance for leading classes, and handouts. See Table 1 for additional information.

**Table 1:** Assessment of prevent T2 curriculum components.

Component	Delivery	Look and Feel	Cultural elements and language	Literacy	Data included
Participant training guide	In person and online delivery available Phase 1—16 sessions delivered weekly to biweekly during months 1–6 Phase 2—6 sessions delivered monthly during months 7–12	Includes table of contents with modules that can be easily accessed by clicking on the appropriate module online Each module includes large, colorful images portraying the goals, title, objectives, examples/stories, worksheets, and bulleted important action steps/points to remember Overall, component is easy to navigate	Includes CDC logo English version incorporates images of foods, people, and activities depicting American culture Spanish version incorporates culturally appropriate examples of food, food measurement, and physical activity with input from native Spanish speakers Available in English and Spanish	English curriculum was written at the 6th-grade reading level Spanish curriculum was written at the 5th-grade reading level Spanish curriculum is not a direct translation but rather a separate, yet similar curriculum developed based on the original Diabetes Prevention Program (DPP) lifestyle intervention trial (44, 52)	Provides data and statistics using language that is easy to understand (e.g., the prevalence of pre-diabetes is presented as “1 in 3 Americans”) Risk factors by age and ethnicity identified, although numbers not provided Chart is provided indicating weight and targets for percent loss
Lifestyle coach training guide	Curriculum can be delivered in-person or online Indicates materials needed for each module. Provides prompts about what the coach can say, do or observe during the sessions.	Guide with 26 modules that can be quickly accessed by clicking on the appropriate module online Includes handouts such as the meeting schedule, fitness log, and weight log Material presented with a white background and black font (for text in the main body) with major headings in blue and sub-headings in green Each module has outline of the activities, page numbers, and time allocated for each session Modules include two columns on each page: (1) Notes to coach; (2) Guided information about the specific activities	Includes the CDC logo Contains words and terminology depicting American culture, such as “pounds” for measurement or “gas” for petrol Provides suggestions for discussions and activities that are tailored to the population Available in English and Spanish	English curriculum was written at the 6th-grade reading level Spanish curriculum was written at the 5th-grade reading level	Does not provide statistics or data explicitly but refers to the additional materials and handouts from the *Prevent T2 curriculum participant training guide*

In examining the logic model of the EBI and comparing it to the logic model of change in Step 1, we identified what *Prevent T2* elements would be maintained and what adaptations would be needed for the new MASLD-specific program—Healthy Liver/Hígado Sano. Most behavioral outcomes and personal determinants were similar between the two logic models, though the health problem of focus differed. With a different health outcome, content related to perceived susceptibility and severity changed to focus on MASLD, including genetic and environmental reasons of Hispanic individuals having higher prevalence of MASLD and long-term implications of not managing MASLD. Similarly, methods and content addressing outcome expectations shifted the focus to the known impact of physical activity, diet, and weight loss on MASLD management, an area of need identified in the interviews.

We also identified the changes needed for program delivery options. For example, face-to-face interactions have been shown to produce greater weight loss compared to web-based delivery (49), and Hispanic adults tend to prefer in-person programs (50). This sentiment was reiterated in our in-depth interviews. Therefore, the adapted curriculum for the MASLD intervention retained the majority of the in-person curriculum from *Prevent T2*. However, a few participants in the in-depth interviews noted that they were familiar and comfortable with the online conferencing platforms that proliferated during the COVID-19 pandemic. They spoke about how such platforms could be useful in the event of transportation issues, lack of childcare options, or adverse weather conditions. Therefore, the proposed MASLD intervention also incorporated the option for synchronous online session delivery into the curriculum.

In correspondence with the CDC, it was noted that the Spanish and English *Prevent T2* modules were not direct translations of each other and that the Spanish version was developed from the original DPP curriculum through a similar, though adjacent process, that included native Spanish-speakers. Therefore, instead of using the English version directly, we decided to retain and back translate the Spanish version of the *Prevent T2* curriculum given our focus on the Hispanic community and the culturally relevant elements (e.g., types of foods, physical activity examples). This would allow us to have a culturally relevant English-language version of the curriculum for Hispanic individuals; for example, we would use familiar names used by the target populations (e.g., Juan) and include depictions of familiar foods (e.g., tortillas, beans, salsa) in the English version. To address the deep structure (cultural factors), we ensured that the curriculum included sections with activities designed for communal settings given the role of *familismo* in the Hispanic population. We also considered deep structure when identifying determinants. When adding additional content, especially content related to MASLD, we planned to align with the 5 to 6th grade reading level of the *Prevent T2* curriculum.

### Step 4. Make adaptations (put into practice)

We adapted the curriculum by revising the content in both the *Prevent T2* Lifestyle Coach and Participant guides. The adaptations reflected information relevant to MASLD management, including statistics on MASLD, risk factors, health outcomes, and management behaviors. Given the similarities in lifestyle behavior changes needed for preventing type 2 diabetes and managing MASLD to prevent disease progression, most of the material on nutrition, physical activity, and weight loss was retained in both guides. The adaptations included changes in the information to reflect the Healthy Liver/Hígado Sano program objectives. The adaptations also included information on limiting alcohol use, which is not currently part of *Prevent T2*. The adapted program (Healthy Liver/Hígado Sano) retained the content-specific scope and sequence used in the *Prevent T2 curriculum,* where 16 core modules were offered, with each model covering a different topic. The Healthy Liver/Hígado Sano curriculum also provided some flexibility to allow Lifestyle Coaches to cover the modules between 4 and 6 months. The curriculum also used warm hues to reflect color patterns used for depictions of the liver, transitioning away from the cold hues used for *Prevent T2*.

**Figure 3:**
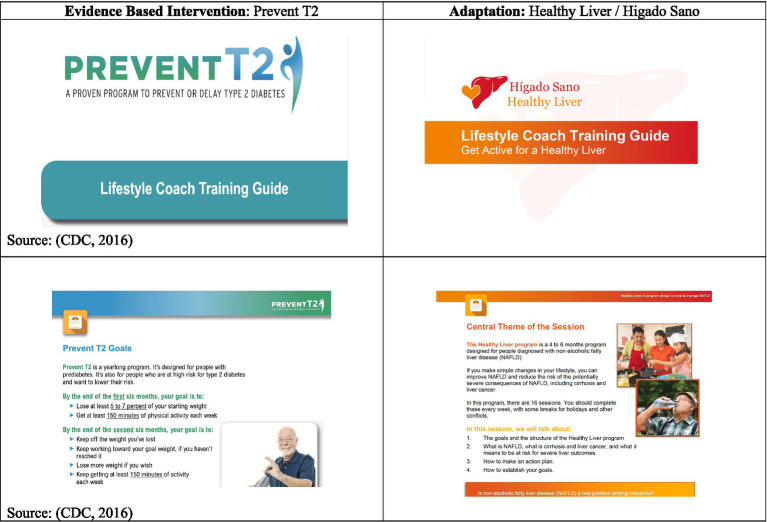
Examples of adaptations: healthy liver/higado sano.

Figure 3 shows examples of pages in the adapted curriculum. Some adaptations made to reflect the MASLD curriculum are outlined in Table 2. Completing Step 4 resulted in final decisions on the delivery setting, timeline of the sessions, intervention content, and other adapted elements.

**Table 2:** Sample adaptations to materials.

Content	Prevent T2 curriculum	Healthy liver program
Logo	“Prevent T2”	“Healthy Liver/Hígado Sano program”
Health problem	Pre-diabetes and Diabetes	NAFLD, fibrosis/cirrhosis and Liver cancer
Weight loss content	“Moderate weight loss can decrease the development of Type 2 diabetes by half.”	“Individuals with a weight loss of at least 5% had a reduction of fat in their liver.”
Information	Content specific to Diabetes, such as insulin, and sugar intake	Content specific to NAFLD, such as explanation of the condition, what is known about disease progression, and reasons for needing to limit alcohol use.
Session 2-Footer	“Get Active to Prevent T2”	“Get Active for a Healthy Liver”

### Step 5. Plan for evaluation

In this paper, we documented the adaptations made to the intervention. A detailed description of the evaluation plan for pilot testing and results of the pilot test will be reported in a subsequent manuscript. Our evaluation will focus on assessing important variables related to the changes, such as disease perceptions and perceived outcome expectations.

## Discussion

Despite the high prevalence of MASLD among Hispanic adults in the United States (2, 3), there is a dearth of EBIs for MASLD management that are culturally relevant for Hispanic adults. MASLD interventions targeting Hispanic adults should consider the health behaviors, the socio-cultural context, and the targeted health outcome. This study described the adaptation process using IM Adapt for the design of the Healthy Liver/Hígado Sano program, a behavioral lifestyle intervention aimed at MASLD management among Hispanic patients. To ensure that we adapted an intervention that was appropriate for our population, we selected an EBI that was feasible, acceptable, and suitable for the target population—the CDC’s DPP curriculum, *Prevent T2* program (46). IM Adapt provided a structured approach for reviewing and adapting other successful programs with similar approaches and goals, eliminating the need to develop a completely new program.

The Healthy Liver/Hígado Sano program is designed to help patients with MASLD improve their healthy eating habits, engage in physical activity, and achieve weight loss to prevent adverse health outcomes related to MASLD disease progression, such as cirrhosis and liver cancer. As an initial step, in-depth interviews with Hispanic patients with MASLD provided perspectives on the utility of and preferences for the proposed intervention. Conducting a needs assessment before program implementation to understand problems affecting the target population is an essential component of the Intervention Mapping approach (22). In our case, this included interviews and discussions directly with the target population members (Hispanic patients with MASLD) where they could share their opinions on a potential behavioral lifestyle intervention, complemented by recent work with the same target population related to lifestyle behaviors (30, 36). This multifaceted needs assessment provided critical information needed to adapt the intervention, such as offering the program primarily in an in-person group format, with flexibility for synchronous online attendance, barriers that needed to be addressed, and providing incentives. However, despite the use of the systematic process, there is still a need to evaluate the adapted intervention to ensure it is feasible and effective.

Few interventions targeting MASLD management have been developed (14), with none tailored to the Hispanic population in the US. IM Adapt provided an iterative planning structure where program planners searched for existing EBIs and adapted the most appropriate intervention for patients with MASLD. The Healthy Liver/Hígado Sano program could be implemented in similar settings within the US or Latin American countries where the prevalence of MASLD is high. However, program implementers should still consider the context of their specific populations and adapt the curriculum accordingly. Importantly, future work could consider the incorporation of social determinants of health into the adaptation process, addressing potential inequities in access to physical activity opportunities and healthy foods (51).

## Conclusion

The Healthy Liver/Hígado Sano program, adapted from the *Prevent T2* program, has the potential to help patients with MASLD manage their condition and lose weight by eating healthy and engaging in physical activity. The needs assessment from the target population (Hispanic patients with MASLD) uncovered potential motivators, barriers, and issues to consider when adapting the intervention. As the program is being implemented and evaluated, the intervention may need to be further adapted based on delivery settings—with the addition and adaptation of implementation strategies specific to the setting.

## Data Availability

The data will be made available by reasonable request to the corresponding author.
